# A novel nanobody-based immunocytokine of a mutant interleukin-2 as a potential cancer therapeutic

**DOI:** 10.1186/s13568-023-01648-2

**Published:** 2024-02-09

**Authors:** Arezoo Beig Parikhani, Rada Dehghan, Yeganeh Talebkhan, Elham Bayat, Alireza Biglari, Mohammad Ali Shokrgozar, Reza Ahangari Cohan, Esmat Mirabzadeh, Soheila Ajdary, Mahdi Behdani

**Affiliations:** 1 Venom and Biotherapeutics Molecules Laboratory, Department of Medical Biotechnology, Biotechnology Research Center, Pasteur Institute of Iran, Tehran, Iran; 2 Department of Medical Biotechnology, Biotechnology Research Center, Pasteur Institute of Iran, Tehran, Iran; 3 School of Medicine, Tehran University of Medical Sciences, Tehran, Iran; 4 National Cell Bank of Iran, Pasteur Institute of Iran, Tehran, Iran; 5 Department of Nanobiotechnology, New Technologies Research Group, Pasteur Institute of Iran, Tehran, Iran; 6 Department of Molecular Medicine, Pasteur Institute of Iran, Tehran, Iran; 7 Department of Immunology, Pasteur Institute of Iran, Tehran, Iran

**Keywords:** Immunotherapy, Immunocytokine, Mutant IL-2, VEGFR2, Nanobody

## Abstract

The immunotherapeutic application of interleukin-2 (IL-2) in cancer treatment is limited by its off-target effects on different cell populations and insufficient activation of anti-tumor effector cells at the site of the tumor upon tolerated doses. Targeting IL-2 to the tumor microenvironment by generating antibody-cytokine fusion proteins (immunocytokine) would be a promising approach to increase efficacy without associated toxicity. In this study, a novel nanobody-based immunocytokine is developed by the fusion of a mutant (m) IL-2 with a decreased affinity toward CD25 to an anti-vascular endothelial growth factor receptor-2 (VEGFR2) specific nanobody, denoted as VGRmIL2-IC. The antigen binding, cell proliferation, IFN-γ-secretion, and cytotoxicity of this new immunocytokine are evaluated and compared to mIL-2 alone. Furthermore, the pharmacokinetic properties are analyzed. Flow cytometry analysis shows that the VGRmIL2-IC molecule can selectively target VEGFR2-positive cells. The results reveal that the immunocytokine is comparable to mIL-2 alone in the stimulation of Primary Peripheral Blood Mononuclear Cells (PBMCs) and cytotoxicity in in vitro conditions. In vivo studies demonstrate improved pharmacokinetic properties of VGRmIL2-IC in comparison to the wild or mutant IL-2 proteins. The results presented here suggest VGRmIL2-IC could be considered a candidate for the treatment of VEGFR2-positive tumors.

## Key Points

- The newly developed IL-2- based immunocytokine may target VEGFR2- positive tumors.
- The fusion of nanobody to IL-2 improved its pharmacokinetics.
- The biological activity of IL-2 was not affected by its genetically fusion to nanobody.

## Introduction

Immunotherapy based on interleukin-2 (IL-2) is an FDA-approved treatment option for metastatic melanoma and renal cell carcinoma (Payne et al. 2014). IL-2 functions as both an immune stimulant and an immune suppressor via binding to its receptor (IL-2R) (Jiang et al. 2016). T lymphocytes and natural killer (NK) cells express intermediate-affinity dimeric IL-2 receptors (IL-2Rβγ), while activated lymphocytes and regulatory T cells (Tregs) express high-affinity trimeric IL-2 receptors (IL-2Rαβγ) (Boyman and Sprent 2012). Besides, CD25 (IL-2Rα) is expressed on endothelial cells (Arenas-Ramirez et al. 2015; Krieg et al. 2010). Following high-dose IL-2 administration, IL-2 binds to this receptor and causes endothelial cell damage which leads to vascular leak syndrome (VLS) (Arenas-Ramirez et al. 2015). Other dose-limiting toxicities of IL-2 treatment include gastrointestinal, neurological, pulmonary, hepatic, renal, and hematological toxicities (Krieg et al. 2010). Furthermore, clinical use of IL-2 has been challenging due to its short serum half-life (Malek and Castro 2010). It has been postulated that the aforementioned limitations will be greatly resolved by the use of a suitable delivery system of IL-2 to the tumor microenvironment to increase efficacy without causing systemic toxicity (Atkins et al. 1999; Davis and Gillies 2003).

One approach for selective cytokine localization at the tumor site is the development of immunocytokine (IC). Immunocytokines are antibody-cytokine fusion proteins with the potential to preferentially localize in the tumor microenvironment (Davis and Gillies 2003; Gutbrodt and Neri 2012). For example, IL-2 is conjugated to an antibody or antibody components, which could bind tumor-associated antigens (TAAs). As a consequence, due to the binding of the antibody to the antigen on the tumor, the local concentration of IL-2 is increased at the tumor site. Furthermore, this approach could improve the half-life of IL-2 and enhance the immune-modulatory effect of this cytokine with less toxicity (Jiang et al. 2016).

Various IL-2-based immunocytokines have been developed during the last two decades. These molecules have shown far better antitumor activity compared with IL-2, the targeting antibody alone, or the combination of both agents (Gillies 2013; Mortara et al. 2018).

Considering the limitations of whole antibodies, such as their high molecular weight and poor penetration within the solid tissues, it seems that antibody fragments could be more effective (Xenaki et al. 2017). Single-domain antibodies or nanobodies (Nb), also referred to as VHH, show a similar structure to the variable region of conventional antibodies (VH). Nb with an approximate molecular mass of 15 kD, first derived from Camelidae, are the smallest naturally occurring antigen-binding fragments. Their high affinity, solubility, stability, ease of production, and low immunogenicity have made them attractive delivery tools in cancer therapy (Buelens et al. 2010).

Development of IC and delivery of cytokines to the tumor site require the presence of specific, accessible, abundant, and stable target antigens, which allow clear discrimination between healthy and tumor cells (Gutbrodt and Neri 2012). Vascular endothelial growth factor receptor-2 (VEGFR2) is an important tumor-associated receptor on the surface of various tumor cells and their surrounding blood vessels. VEGFR2 has been suggested as a promising target for cancer treatment due to its high expression on lung, colorectal, ovarian, urothelial, prostate, head and neck, cervix, and skin cancer cells (Donnem et al. 2007; Giatromanolaki et al. 2007; Lu et al. 2019; Spannuth et al. 2009).

We have previously developed an anti-VEGFR2 nanobody with high affinity and specificity, referred to as 3VGR19 (Behdani et al. 2012). This nanobody, which binds human VEGFR2 with a *KD* value of 5.4 nM, has been isolated from a hyper-immunized camel library and expressed solublely in *E. coli* WK6 cells. 3VGR19 could selectively recognize VEGFR2-overexpressing tumor cells and potently inhibit the formation of capillary-like structures. Besides, recently, we introduced a mutant IL-2 (mIL-2) based on a decrease in electrostatic interactions between IL-2 and IL-2Rα to preferentially stimulate cytotoxic CD8+ T and NK cells while not affecting Tregs (Dehghan et al. 2022). This new mIL-2 demonstrated less affinity for the IL-2R but more effective cytotoxicity and antitumor activity compared to the wild-type IL-2 (wtIL-2) (Beig Parikhani et al. 2022; Dehghan et al. 2022). In the present study, with the aim of targeted delivery of mIL-2 to VEGFR2-overexpressing tumor cells, an immunocytokine (VGRmIL2-IC) compromising the previously developed mIL-2 and 3VGR319 was designed and expressed in *E. coli*, and its characteristics were evaluated.

## Materials and methods

### Animals and cells

Female BALB/c mice (6–8 weeks old) were purchased from the Animal Center of the Pasteur Institute of Iran (Karaj, Iran). The research protocols and all animal studies were approved by the Ethics Committee of the Pasteur Institute of Iran (IR.PII.REC.1400.034) and followed ARRIVE reporting guidelines (Percie du Sert et al. 2020). HEK293 and 293KDR cell lines were obtained from the National Cell Bank of Iran (Pasteur Institute of Iran, Tehran, Iran). 293KDR is a stably transfected cell line with high expression of VEGFR2. Human Peripheral Blood Mononuclear Cells (PBMCs) were isolated from heparinized blood samples taken from healthy donors using Ficoll-Hypaque (Lymphodex, Innotrain, Germany) density gradient centrifugation according to the manufacturer’s protocol.

### Immunocytokine expression and purification

Anti-VEGFR2 nanobody (Behdani et al. 2012), and mIL2 (Beig Parikhani et al. 2022) gene fragments were linked via the hinge polypeptide of Llama IgG2c as a linker and subcloned in the pET28a expression vector (ShineGene, China) possessing a C-terminal Histidine tag.

The designed recombinant immunocytokine (VGRmIL2-IC), mIL-2, and wtIL-2 proteins were expressed in *E. coli* BL21 (DE3) using 0.5 mM IPTG for 6 h at 37 °C. Finally, *E. coli* cells were pelleted, and the supernatants were discarded.

For protein purification, the bacterial pellets were suspended in lysis buffer containing 10 mM imidazole, 0.5 M NaCl, and 50 mM NaH_2_PO_4_, pH 8.0, and sonicated on ice for 10 min. The suspension was centrifuged at 10,000×*g* for 20 min. The pellet containing the inclusion bodies (IB) was solubilized in a solubilization buffer (8 M urea, 50 mM NaH_2_PO_4_, 300 mM NaCl, and 10 mM imidazole) and incubated for 1 h at room temperature. After centrifugation (10,000×*g* for 30 min), the supernatant was clarified through a 0.45 μm membrane filter, followed by applying to the Ni-NTA resin (ABT company) at a flow rate of 1 ml/min. Gradual removal of urea (on-column refolding) was performed during purification by refolding buffer (50 mM NaH_2_PO_4_, 0.5 M NaCl, 20 mM Imidazole, pH 8.0, containing urea from 8 to 0 M). 0.1% Triton X-114 was used within the first washing buffer in order to remove bacterial LPS in affinity chromatography. Elution and recovery of the captured His-tagged proteins were accomplished by using a high concentration of imidazole buffer (250 mM imidazole, 50 mM NaH_2_PO_4_, 0.5 M NaCl; pH 8.0). Finally, the LPS level of the purified protein was quantified by the Pyrotell gel clot LAL kit (USA; sensitivity 0.25 EU/ml of analyzed solution) according to the manufacturer’s instruction.

The purity of the eluted proteins was tested on 12% SDS-PAGE and Coomassie Brilliant Blue staining. Furthermore, protein identification was verified by western blotting. In brief, after electrophoresis, the proteins were transferred onto the PVDF membrane using the semi-dry transfer system (Bio-Rad, USA). The membrane was blocked (3% w/v skim milk in PBS) for 2 h at room temperature and incubated overnight with rabbit anti-Histidine primary antibody produced in our laboratory. The membrane was then incubated with goat anti-rabbit HRP-conjugated secondary antibody (Sigma, USA) for 4 h. Finally, the protein bands were visualized by the 3,3′-diaminobenzidine (DAB) substrate solution. After confirmation of the proteins’ purity, we concentrated and filtered the protein with 0.22 μm syringe filters before in vitro and in vivo tests.

### Antigen binding assay

The binding capability of VGRmIL2-IC towards VEGFR2 was evaluated by flow cytometry using 293KDR and HEK293 cell lines as VEGFR2 positive and negative cell lines, respectively. In brief, the 293KDR or HEK-293 cells were cultured in DMEM medium supplemented with 10% fetal bovine serum (FBS, Sigma) at 37 °C under 5% CO_2_ atmosphere. Then, the harvested cells were centrifuged and resuspended in PBS supplemented with 3% FBS (FACS buffer). 1 × 10^6^ cells/ml were incubated with 1 µg of 3VGR-319 Nb or equimolar amounts of VGRmIL2-IC for 1 h on ice. After washing with FACS buffer, a homemade rabbit anti-camel antibody was added to each tube and incubated for a further 1 h, which was followed by twice washing with FACS buffer and incubation with FITC-conjugated anti-rabbit IgG antibody (Abcam, UK) for 30 min. In this assay, the commercial PE anti-human VEGFR2 antibody was used as a reference. The signals were detected using a Partec PAS III flow cytometer (Partec GmbH, Germany), and analysis was performed using FlowJo software (Tree Star, Inc., USA).

### IL-2 bioactivity assay

The proliferative activity of IL-2-based immunocytokine was evaluated in a standard T-cell proliferation assay using concanavalin A (ConA)-stimulated PBMCs, which highly express trimeric IL-2R (Thornton and Shevach 1998). Proliferation was measured by the reduction of the Alamar Blue cell viability reagent. PBMCs were washed with sterile PBS and cultured in RPMI medium supplemented with 10% FBS, 2 mM l-glutamine, 100 U/ml penicillin, and 100 µg/ml streptomycin as a growth medium (GM), and 5 µg/ml ConA was added. After 24 h of incubation, the cells were washed and incubated for another 24 h in ConA-free GM. The starved cells (2 × 10^5^ cells/well) were seeded into 96-well plates in 200 µl of GM containing different equimolar concentrations of VGRmIL2-IC or mIL-2 (as control) for 48 h, followed by the addition of 10% Alamar Blue to each well. The cells were incubated for a further 12 h, and optical density was analyzed by absorbance measurements at 570 and 600 nm. The reduced percentage of Alamar Blue was calculated according to the manufacturer’s recommendations (Thermo Fisher Scientific, USA). Finally, the results were fit to sigmoidal concentration-response curves with four-parameter logistic regression (4PL), and EC50 was calculated.

### IFN-γ secretion

Freshly isolated PBMCs (1 × 10^6^ cells/well) were seeded into the 48-well plate. Stimulation with a distinctive equimolar concentration of VGRmIL2-IC and mIL-2 was performed in triplicate. After 48 h of incubation at 37 °C in a humidified 5% CO_2_ atmosphere, the supernatants were collected, and the secreted IFN-γ levels were measured using a human IFN-γ ELISA kit (Karmania Pars Gene, Iran).

### Cytotoxicity assay

The calcein-AM release method was used for the cytotoxicity assay. The target 293KDR cell line (TC) was labeled with calcein-AM (R&D Systems, USA) and co-incubated with freshly isolated PBMCs as the effector cells (EC) at various EC:TC ratios of 40:1, 20:1, 10:1, and 1:1 without or with VGRmIL2-IC, wtIL-2, and mIL-2 for 16 h. The maximum and spontaneous release controls were cells treated with 2% Triton X-100 and plain media, respectively. After incubation, the plate was centrifuged at 250 g for 2 min, and 150 µl of the supernatants were transferred into the black 96-well plate (Thermo Scientific, USA), and the calcein signal was measured using the BioTeK fluorometer at 485 and 530 nm as excitation and emission wavelengths, respectively. Finally, the lysis percentage of each group was calculated using the following formula: [Test release-spontaneous release/Maximum release-spontaneous release] × 100.

### Pharmacokinetic study

Female BALB/c mice (18–20 g, n = 3) received a single intravenous injection of (0.8 mg/kg) wtIL-2, mIL-2, or VGRmIL2-IC in a volume of 0.2 ml. Blood samples were collected at different time intervals (0, 15 min, 30 min, 1 h, 2 h, 4 h, 8 h, 12 h, 24 h, and 48 h), and the sera were stored at − 20 °C in triplicate. Finally, the concentrations of IL-2 and VGRmIL2-IC were evaluated using the human IL-2 Quantikine ELISA Kit (R & D Systems, U.S). The pharmacokinetic parameters, including the area under the curve (AUC) from time zero to the time that protein is detectable in the circulation (AUC 0–t), AUC 0–∞, terminal half-life (t½), elimination rate constant, mean residence time (MRT), and apparent total clearance rate (CL/F), were calculated for wtIL-2, mIL-2, and VGRmIL2-IC.

### Statistical analysis

Graph plotting and statistical analysis were performed using GraphPad Prism software (v. 8.0) (GraphPad Software, U.S.). A one-way ANOVA test was applied to compare the mean values of the studied parameters, and *P* values less than 0.05 were considered statistically significant.

## Results

### Protein production

**Fig. 1:**
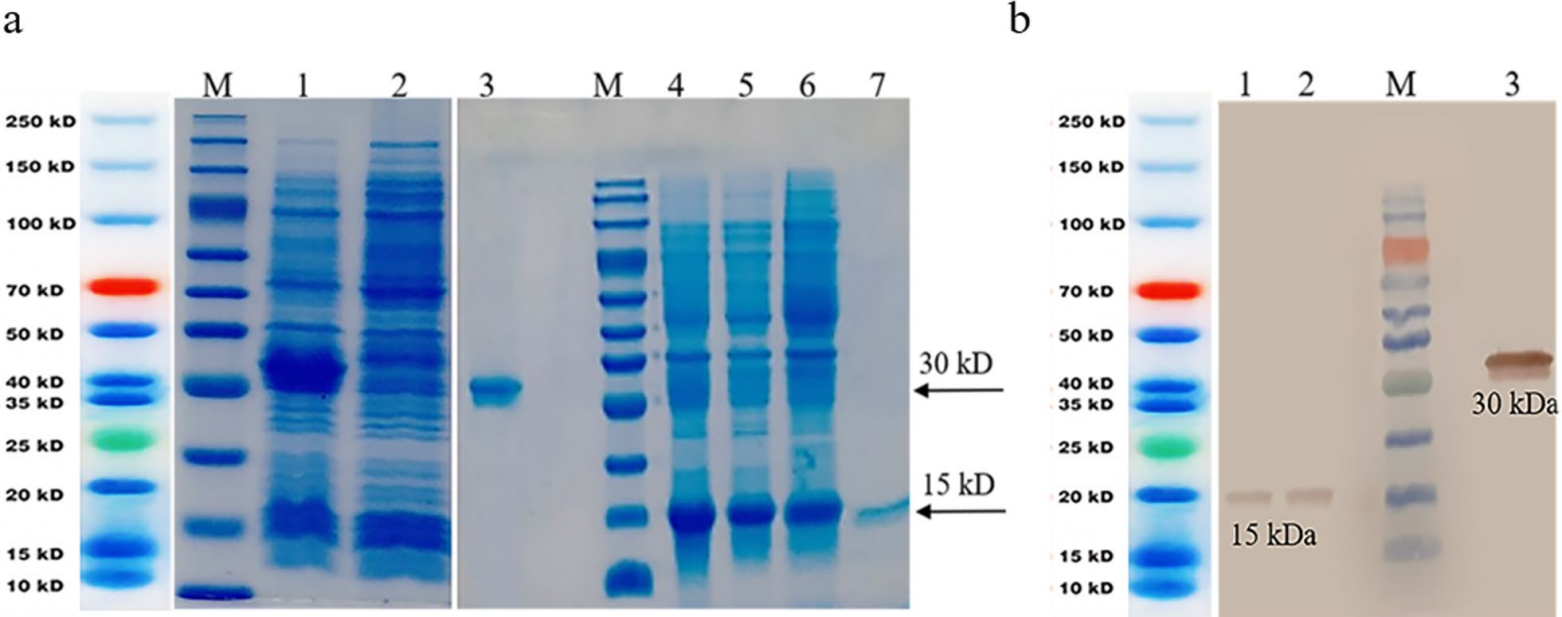
SDS-PAGE and western blotting analysis of protein expression **a** SDS-PAGE: M: protein molecular weight marker; #1, 2: Lysates of *E. coli* cells after and before induction of VGRmIL2-IC expression #3: Purified VGRmIL2-IC protein; #4: Lysate of *E. coli* cells after induction of wtIL-2 expression; #5, 6: Lysate of *E. coli* cells after induction of mIL-2 expression; 7: Purified mIL-2 **b** Western blotting analysis: #1; Purified wtIL-2; #2: Purified mIL-2; #3: Purified VGRmIL2-IC

All 3 proteins (wtIL-2, mIL-2, and VGRmIL2-IC) were expressed in BL21 DE3 bacterial host cells by the IPTG inducer in the form of IB. 12% SDS-PAGE and western blotting confirmed the expression and purification of VGRmIL2-IC, wild, and mutated IL-2 proteins with a molecular weight of about 30 and 15 kDa, respectively (Fig. 1). Moreover, LPS content of the purified protein was lower than the detection limit of the kit.

### VGRmIL2-IC can specifically bind to VEGFR2-positive cells

**Fig. 2:**
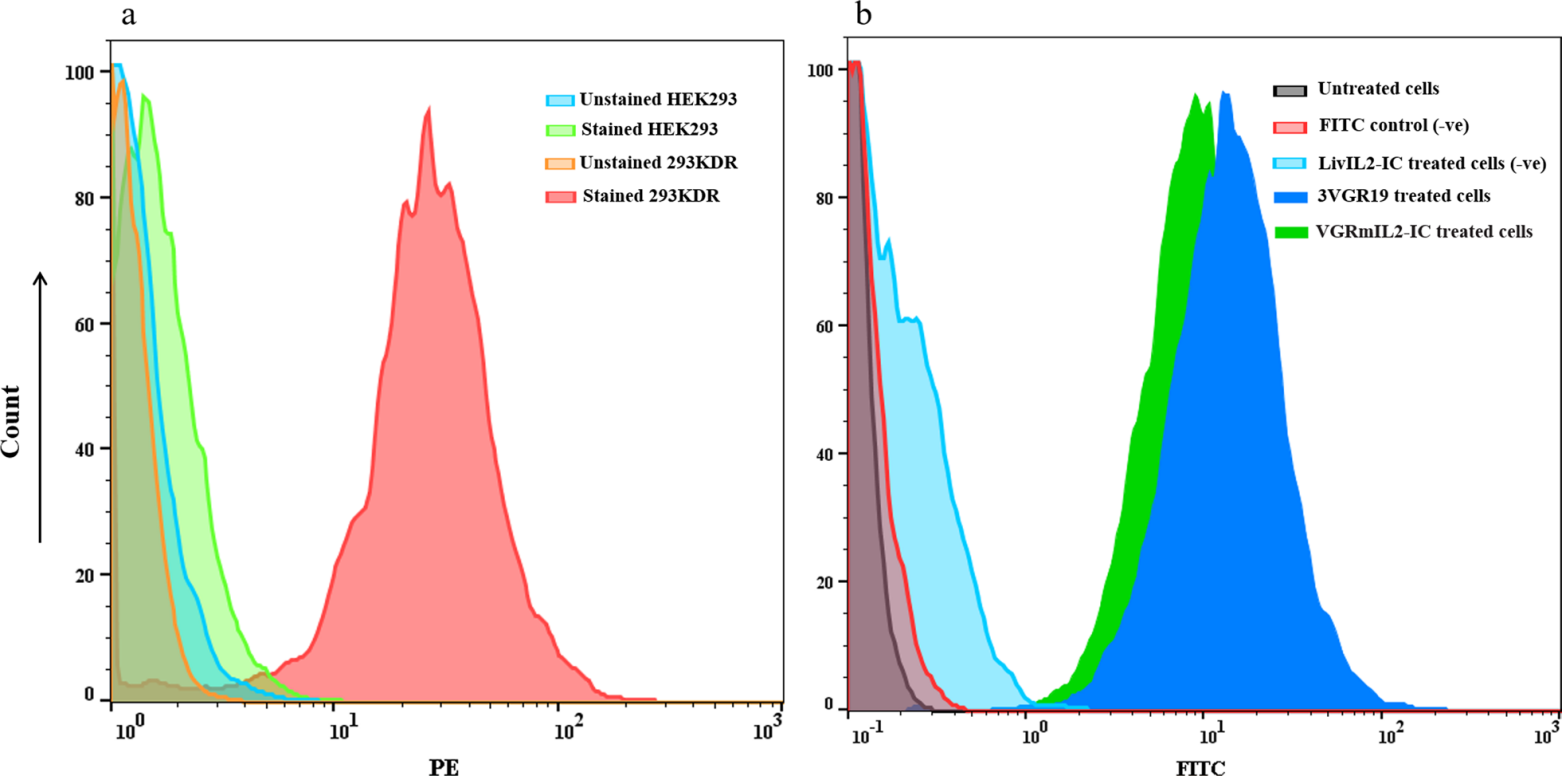
Flow cytometry analysis **a** VEGFR2 expression analysis on the surface of 293KDR and HEK-293 cells using commercially available anti-VEGFR2 PE-conjugated antibody: Blue and green graphs: Unstained and stained HEK-293 cells, respectively; Orange and red graphs: Unstained and stained 293KDR cells, respectively. **b** Binding potency analysis of VGRmIL2-IC and 3VGR19 Nb towards 293KDR cells using a homemade rabbit anti-camel antibody followed by FITC-conjugated anti-rabbit IgG antibody, gray graph: Untreated control cells; Red graph: Secondary antibody alone control; Light blue graph: LivIL2-IC as a non-specific Nb-IL2 control; Dark blue and green graphs: 3VGR19 Nb and VGRmIL2-IC treated cells, respectively

Flow cytometry analysis was performed to assess the binding capacity of the recombinant immunocytokine. A commercial anti-VEGFR2 antibody was used to confirm the expression of VEGFR2 on 293KDR cells. A strong positive signal (84%) was observed in comparison with unstained 293KDR cells and stained HEK293 as negative controls (Fig. 2a). Analysis of the binding capacity of VGRmIL2-IC and 3VGR19 towards 293KDR cells revealed that at equal concentrations, the mean fluorescence intensities (MFI) were 11.4 and 17.7 for VGRmIL2-IC and 3VGR19 treated cells, respectively (Fig. 2b).

### VGRmIL2-IC behaves similarly to mIL-2 in bioactivity

**Fig. 3:**
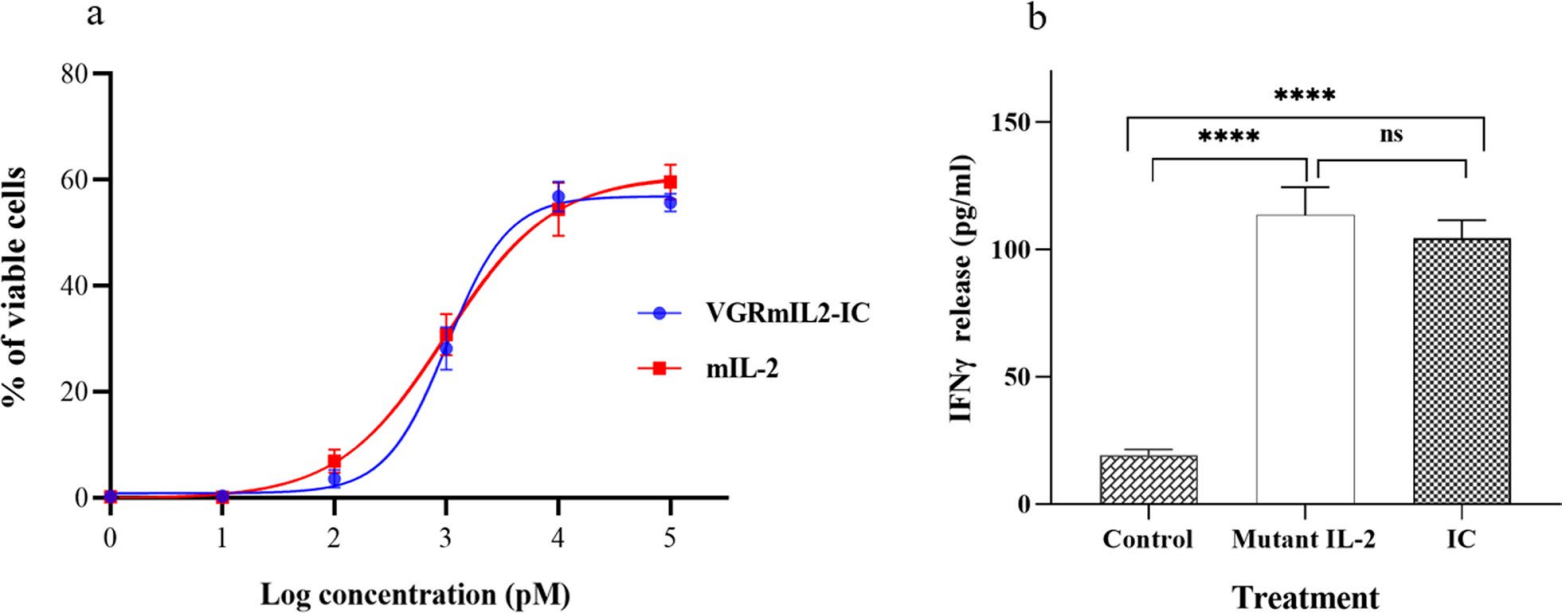
**a** PBMC proliferation assay analysis by Alamar Blue reduction. Starved ConA-activated PBMCs were stimulated with different equimolar concentrations (1–105 pM) of VGRmIL2-IC or mIL-2 for 48 h. **b** IFN-γ secretion analysis by ELISA assay. PBMCs were stimulated with 30 pmol of VGRmIL2-IC or mIL-2. After 48 h of incubation, the supernatants were collected, and the secreted IFN-γ levels were measured. The results are the mean ± SD values of triplicate experiments. Data analysis was performed using a one-way ANOVA test (ns: not significant; ****P < 0.0001)

A cell proliferation assay was performed to compare the bioactivity of VGRmIL2-IC and mIL-2 proteins. A comparison of calculated logEC50 values (2.975 and 3.009 for mIL-2 and VGRmIL2-IC, respectively) indicated that the EC50 values of the two tested IL-2 derivatives had no significant difference (*P > 0.05*) (Fig. 3a).

### IFN-γ secretion is increased in response to IL-2 derivatives

The effect of immunocytokine on IFN-γ secretion was compared to that of mIL-2. PBMCs were stimulated with VGRmIL2-IC or mIL-2 for 48 h, and IFN-γ levels in the supernatants were measured by ELISA. In comparison to control cells that received no treatment, IFN-γ secretion was significantly increased in response to VGRmIL2-IC and mIL-2. The two IL-2 derivatives did not show any significant difference in IFN-γ secretion (Fig. 3b).

### IL-2 derivatives have a higher cytotoxicity effect than wtIL-2

**Fig. 4:**
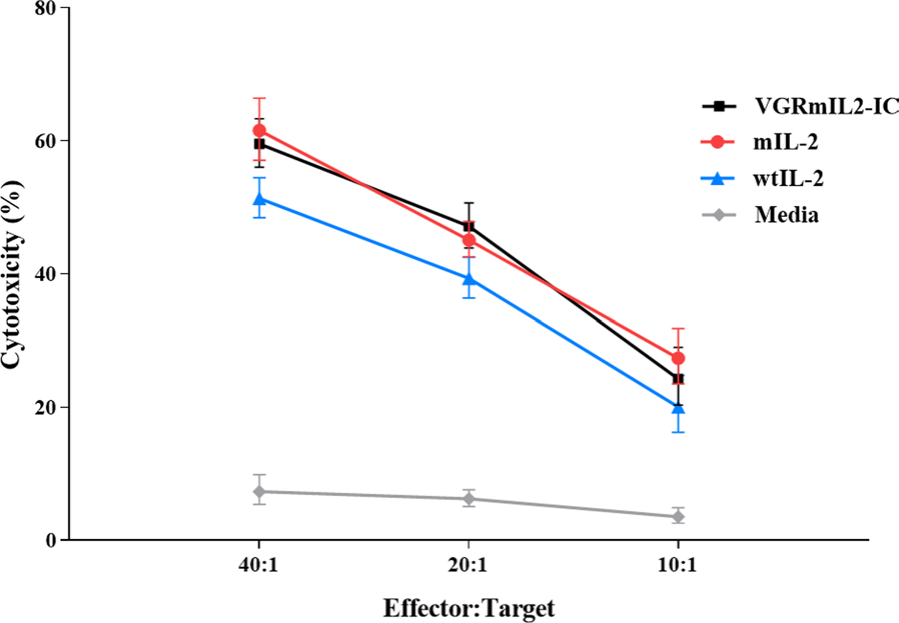
Cellular cytotoxicity assay at different effector (PBMCs) and target cells (293KDR) ratios. Untreated PBMCs without exogenous cytokines served as control. Experiments were repeated three times and the results are presented as mean ± SD. Multiple comparisons were performed using 2-way ANOVA

293KDR target cells (TC) were labeled with calcein and co-cultured with different ratios of PBMCs (the effector cells) in the presence or absence of VGRmIL2-IC, mIL-2, or wtIL-2 proteins. The measured calcein fluorescence signal from the culture supernatants demonstrated increased cytotoxicity in all EC:TC ratios that were treated with the three IL-2 derivatives compared with untreated control cells (*P* > 0.05). It was also observed that higher EC:TC ratios were correlated with stronger cytotoxicity effects. Furthermore, VGRmIL2-IC and mIL-2 proteins induced significantly higher cytotoxicity in comparison to the wtIL-2-treated cells (*P >* 0.0001) (Fig. 4).

### Fusion of 3VGR19 to mIL-2 improved pharmacokinetic properties

**Fig. 5:**
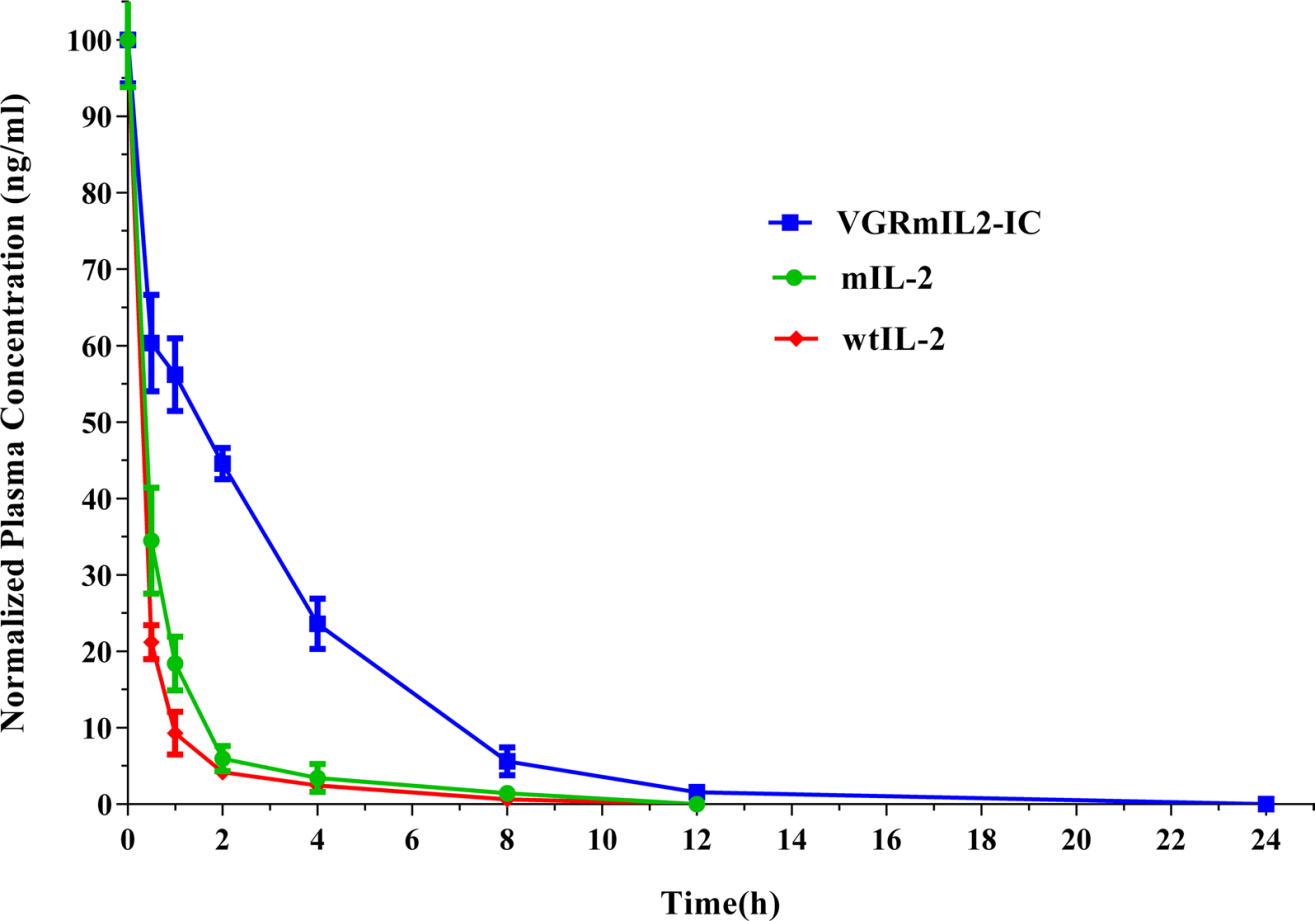
Pharmacokinetic profiles of VGRmIL2-IC, wtIL-2, and mIL-2 in mice. Monitoring of wtIL-2 and mIL-2 was performed for up to 48 h. Cytokines were not detectable in the plasma after 24 h

Serum levels of wtIL-2, mIL-2, and VGRmIL2-IC were determined within 48 h following intravenous administration to the mice. Figure 5 shows the pharmacokinetic data obtained from 3 mice for each group. A pharmacokinetic study revealed that the terminal half-life (t½) and the AUC of VGRmIL2-IC significantly increased compared to mIL-2 and wtIL-2. The plasma clearance curve of VGRmIL2-IC was different from mIL-2 and wtIL-2. The pharmacokinetic parameters summarized in Table 1 showed that the MRT of mIL-2 and wtIL-2 differ slightly, while the MRT of VGRmIL2-IC showed a ~ 2.2-fold and 1.75-fold increase when compared to wtIL-2 and mIL-2, respectively. The lowest clearance rate was obtained for VGRmIL2-IC which was 2.3- and 4.1-fold lower than mIL-2 and wtIL-2, respectively (Table 1).

**Table 1:** Summary of calculated pharmacokinetic parameters using a log-linear trapezoidal method for VGRmIL2-IC, wtIL-2, and mIL-2 following intravenous administration in mice

Proteins	AUC_(0–t)_ (ng. h/ml)	AUC_(0–∞)_ (ng. h/ml)	t_1/2_ (h)	CL/F (ml/h/kg)	K_e_ (h^−1^)	MRT (h)
VGRmIL2-IC	4361.954	4459.913	2.83	0.67	0.245	2.78203
wtIL-2	947.7558	977.9066	1.74	2.78	0.398	1.219605
mIL-2	1304.598	1331.933	1.58	1.57	0.439	1.541295

## Discussion

IL-2 is one of the first approved cytokines for cancer immunotherapy. However, its clinical application has generally been accompanied by severe side effects, which could be due to its widely expressed receptors on the surface of endothelial and immune cells (Chen et al. 2016). Through potential targeted delivery of cytokines, it is possible to obtain decreased side effects and an improved therapeutic index by concentrating the immune stimulators at the tumor site (Gillies 2013; Runbeck et al. 2021). In the present study, an immunocytokine comprising the 3VGR19 nanobody and a mutated IL-2 that possessed three mutations corresponding to a reduced CD25 binding property was successfully expressed in the *E. coli* BL21 (DE3) host strain with the hope of increasing efficacy and decreasing the associated toxicity.

Evaluation of antigen binding potency by flow cytometry analysis demonstrated that the developed immunocytokine could specifically recognize and bind to VEGFR2, similar to 3VGR19. It has been shown that selectively binding immunocytokines to target antigens on tumor cells focus their effects on the tumor microenvironment and facilitates tumor cell lysis by IL2R+ T cells and NK cells (Gillies et al. 1992; Hank et al. 1996; Sondel and Gillies 2012). Prostate-specific membrane antigen (PSMA), programmed death ligand-1 (PD-L1), EGFR, carbonic anhydrase IX (CAIX), and CD20 are just a few of the antigens that IL-2 immunocytokines have been created to target. This means that these immunocytokines that specifically target cancer cells have a wider therapeutic window than unconjugated IL-2 (Chen et al. 2016; Christ et al. 2001; Dougan et al. 2018; Gillies et al. 2005; Runbeck et al. 2021; Sugimoto et al. 2014; Ziffels et al. 2019).

In a previous study, we demonstrated that mIL-2 has a poorer proliferation response than wtIL-2 (Beig Parikhani et al. 2022). Analysis of the biological activity of the IL-2 moiety in the proliferation assay of PBMCs revealed that VGRmIL2-IC and mIL-2 have comparable responses. This finding is in agreement with previous reports showing that fusion proteins did not significantly affect the activity of the IL-2 moiety (Gubbels et al. 2011). It appears that the high-affinity binding of IL-2 to the receptor correlates with its proliferative responsiveness, and weaker interaction between IL-2 and the α subunit may result in a reduction in the cytokine’s proliferative activity (Carmenate et al. 2018; Heaton et al. 1993).

Furthermore, previous studies have indicated that the interaction of IL-2 with high-affinity IL-2R on human PBMCs and activation of several cell types within blood circulation through the intermediate IL-2R can lead to the maximal secretion of proinflammatory cytokines such as TNF-α, IL-1, and IFN-γ, which are believed to mediate the toxicity associated with IL-2-based immunotherapies (Baluna and Vitetta 1997; Siegel and Puri 1991). We examined the cellular IFN-γ secretion level induced by VGRmIL2-IC compared to that of mIL-2. The findings revealed that IFN-γ secretion by PBMCs following VGRmIL2-IC treatment was comparable to that of mIL-2-treated cells. We have previously shown that the induction of IFN-γ by mIL-2 was lower than that of wtIL-2 (Beig Parikhani et al. 2022). Numerous studies have reported that a reduction in secondary cytokine release would ameliorate IL-2 toxicity without affecting its antitumor efficacy (Edwards et al. 1992; Gubbels et al. 2011; Heaton et al. 1993). Therefore, it seems that the developed immunocytokine could be an effective and less toxic means of cancer treatment with reduced affinity for the α-subunit of IL-2R.

According to the results of the cytotoxicity evaluation, VGRmIL2-IC and mIL-2 showed stronger cytotoxic effects by PBMC against 293KDR cells possessing VEGFR2 compared to wtIL-2. Additionally, their cytotoxic activity rises with greater effector-target ratios. Stronger cytotoxic activity in the presence of VGRmIL2-IC could be the result of the bispecific nature of the immunocytokine molecule, which leads to stronger adhesion of effector cells to the target ones. Moreover, we have previously shown the cytotoxic effect of our mIL2 on the co-culture of PBMC with the target of NK cells, i.e., K562 (Dehghan et al. 2022). These results indicate the involvement of NK cells as an effector cell.

The huKS-IL2 IC improves NK-tumor cell conjugation by exclusively targeting the EpCAM antigen expressed in lung, ovary, colon, and other adenocarcinomas (Buhtoiarov et al. 2011; Connor et al. 2004; Kim et al. 2009). Likewise, hu14.18-IL2 IC, which recognizes the GD2 disialoganglioside expressed on human neuroectodermally derived tumors, induces conjugation between NK or RL12 cells and the GD2+ tumor cells, resulting in IC-mediated cytotoxicity (Buhtoiarov et al. 2011).

IL-2 is known to be cleared rapidly from circulation. Previous studies have shown that due to the short serum half-life, the administration of higher doses of IL-2 will be required for efficient antitumor activity, which will lead to unacceptable toxicity in treated cancer patients (Konrad et al. 1990; Tang and Harding 2019). The fusion of IL-2 with another protein moiety can be assumed to be a successful approach to improving the in vivo half-life (Kontermann 2011). The pharmacokinetic behavior of VGRmIL2-IC was evaluated after an i.v. injection into BALB/c mice. The results indicated that the plasma concentration-time profile of VGRmIL2-IC protein was wider and the AUC (0–t) value was higher than those of wtIL-2 and mIL-2. These findings indicate the improved plasma circulation of VGRmIL2-IC compared to mIL-2 and wtIL-2. Furthermore, the fusion of 3VGR19 with mIL-2 improved the circulatory half-life (t½) of this cytokine in mice. This increase could be the result of increasing the size of protein, therefore reducing its clearance by the kidney. In designing ICs, in addition to the choice of cytokine and the target molecules on tumor cells, pharmacokinetics should be considered to improve the efficacy of the IC and the patient’s convenience.

To the best of our knowledge, this is the first study reporting an immunocytokine produced by genetic conjugation of a mutated IL-2 with a reduced affinity towards CD25 to a nanobody. The results of the present study indicated an improved pharmacokinetic of VGRmIL2-IC compared to those of mIL-2 and wtIL-2. In addition, the VGRmIL2-IC enhances the anti-tumor cytotoxic effect while conserving the proliferative and IFN-γ induction properties of IL-2. These preliminary data represent VGRmIL2-IC as a putative immunocytokine for cancer therapeutic approaches. We have previously shown the specific binding of our anti-VEGFR2 nanobody to 293KDR and HUVECs (positive cell lines) and its ability to inhibit capillary-like structure formation (Behdani et al. 2012). Moreover, the effects of mIL-2 on the stimulation of PBMCs to kill K562 and its anti-tumor activity against TC-1 cells in C57BL/6 mice were documented (Dehghan et al. 2022). However, since we did not have access to the VEGFR2-expressing tumor model, the tumor-targeting and immunotherapeutic behaviors of VGRmIL2-IC were not explored in vivo. Furthermore, even though the behavior of wtIL-2 and mIL-2 was investigated in the previous study (Dehghan et al. 2022), there is a need to design an immunocytokine containing wtIL-2 (VGRwtIL2-IC) and compare it with the VGRmIL2-IC designed in this study. The in vivo effect of this new immunocytokine remains to be studied.

## Data Availability

All data supporting the findings of this study are available within the paper.

## Abbreviations

ConA: Concanavalin A
FDA: Food and Drug Administration
IB: Inclusion body
IL-2: Human interleukin-2
IL-2R: Interleukin-2 receptor
IPTG: Isopropyl β-d-1-thiogalactoside
mAb: Monoclonal antibody
PBMC: Peripheral blood mononuclear cell
SDS-PAGE: Sodium dodecyl sulfate polyacrylamide gel electrophoresis
TNF: Tumor necrosis factor
IFN-γ: Interferon gamma
IL-1: Interleukin-1
Treg: Regulatory T cell
NK: Natural killer cell
VLS: Vascular leak syndrome
wtIL-2: Wild-type human interleukin-2
IC: Immunocytokine
PK: Pharmacokinetic
Calcein-AM: Calcein-acetoxymethyl ester
AUC: Area under plasma concentration
